# Retraction Note: In vitro evaluation of the toxic effects of MgO nanostructure in Hela cell line

**DOI:** 10.1038/s41598-023-40237-y

**Published:** 2023-08-09

**Authors:** M. Waseem Akram, Muhammad Fakhar-e-Alam, M. Atif, Alvina Rafique Butt, Ali Asghar, Yasir Jamil, K. S. Alimgeer, Zhiming M. Wang

**Affiliations:** 1 Institute of Fundamental and Frontier Science, University of Electronic Science and Technology of China, Chengdu, 610054 China; 2 Department of Physics, Government College University, Faisalabad, 38000 Pakistan; 3 Department of Physics and Astronomy, College of Science, King Saud University, Riyadh, Saudi Arabia; 4 National Institute of Laser and Optronics, Nilore, Islamabad, Pakistan; 5 Physics Department, Government College University (GCU), Lahore, Pakistan; 6 Department of Mathematics and Statistics, University of Lahore, Lahore, Pakistan; 7 Laser Spectroscopy Lab., Department of Physics, University of Agriculture Faisalabad, Faisalabad, Pakistan; 8 COMSATS Institute of Information Technology, Islamabad, Pakistan

Retraction of: *Scientific Reports* 10.1038/s41598-018-23105-y, published online 15 March 2018

The Editors have retracted this Article.

After publication, concerns were raised regarding potentially overlapping images with other articles published by some of the same authors. Specifically:

- Figure 12B, C and D appear to have been published previously in 2C, 7B, C and D of^1^ but described as a different cell line;
- Figure 12C appear to have been also published previously in 3D of^2^ and also described as a different cell line.

The Authors were unable to provide a satisfactory explanation for this overlap. The Editors therefore no longer have confidence in the validity of the data and the conclusions drawn.

M. Waseem Akram, Ali Asghar, K. S. Alimgeer, Zhiming M. Wang agreed to the retraction and its wording. Yasir Jamil has not confirmed whether they agree or disagree with the retraction. Muhammad Fakhar-e-Alam, M. Atif, and Alvina Rafique Butt did not respond to the correspondence about the retraction.
